# Messung von Patient:innenerfahrungen im Gesundheitswesen – Methodenüberblick und der Fragebogen zur erlebten Patient:innenorientierung (EPAT) als Beispiel

**DOI:** 10.1007/s00103-026-04186-x

**Published:** 2026-01-12

**Authors:** Eva Christalle, Fabia von Blücher, Isabelle Scholl

**Affiliations:** Institut und Poliklinik für Medizinische Psychologie, Universitätsklinikum Hamburg-Eppendorf, Martinistraße 52, 20246 Hamburg, Deutschland

**Keywords:** Patient:innenzentrierung, Patient:innenberichtete Erfahrungsmaße (PREMs), Messung, Versorgungsqualität, Fragebogenentwicklung und -evaluation, Patient focus, Patient-reported experience measures (PREMs), Measurement, Healthcare quality, Questionnaire development and evaluation

## Abstract

Patient:innenorientierung ist ein zentrales Qualitätsmerkmal moderner Gesundheitsversorgung. Um diese systematisch zu erfassen, werden international sogenannte Patient Reported Experience Measures (PREMs) eingesetzt. Sie ermöglichen eine standardisierte Messung von Patient:innenerfahrungen und liefern wichtige Informationen zur Bewertung und Verbesserung der Versorgung. In Deutschland fehlt bislang ein flächendeckender, systematischer Einsatz solcher Instrumente.

Mit dem „Fragebogen zur erlebten Patient:innenorientierung“ (EPAT) liegt nun erstmals ein generisches PREM in deutscher Sprache vor, das Patient:innenorientierung umfassend misst. Der EPAT wurde auf Basis eines umfassenden, methodisch fundierten Entwicklungsprozesses erstellt und erfasst alle relevanten Dimensionen von Patient:innenorientierung. Er weist eine hohe Inhaltsvalidität sowie gute psychometrische Eigenschaften auf. Durch die Verfügbarkeit einer Lang- und Kurzversion ist er flexibel in unterschiedlichen Versorgungskontexten einsetzbar – sowohl in der ambulanten als auch in der stationären Versorgung.

PREMs wie der EPAT eignen sich für vielfältige Einsatzbereiche, z. B. zur Versorgungsforschung, als Feedback-Instrument oder zum Vergleich in Benchmarkings. Der flächendeckende Einsatz von PREMs und eine transparente Berichterstattung der Ergebnisse werden sowohl von der Politik gefordert als auch von Patient:innen gewünscht. PREMs finden zunehmend Einsatz in der Qualitätssicherung und der Effekt ihres Einsatzes wird zunehmend wissenschaftlich untersucht. Insgesamt birgt der Einsatz von PREMs großes Potenzial für eine zukunftsorientierte, patient:innenzentrierte Gesundheitsversorgung.

**Zusatzmaterial online:**

Zusätzliche Informationen sind in der Online-Version dieses Artikels (10.1007/s00103-026-04186-x) enthalten.

## Abstract (en)

Patient-centeredness is a key quality indicator of modern healthcare. To systematically assess this aspect, patient-reported experience measures (PREMs) are increasingly used internationally. These tools enable standardized measurement of patient experiences and provide valuable information for evaluating and improving healthcare services. In Germany, however, there is still a lack of widespread, systematic use of such instruments.

With the Experienced Patient-Centeredness Questionnaire (EPAT), a generic PREM is now available in German for the first time that comprehensively measures patient-centeredness. Developed through a rigorous and methodologically sound process, the EPAT captures all relevant dimensions of patient-centeredness. It demonstrates high content validity and strong psychometric properties. Its availability in both long and short versions allows for flexible use across various care settings—in both outpatient and inpatient contexts.

PREMs like the EPAT are suitable for a wide range of applications, including health services research, as feedback instruments, or for benchmarking purposes. A broad implementation of PREMs and transparent reporting of results are called for by policymakers and demanded by patients. PREMs are gaining importance in quality assurance, and their impact is being increasingly explored in scientific studies. Overall, the use of PREMs holds significant potential for a forward-looking, patient-centered healthcare system.

## Hintergrund

Um Patient:innenorientierung (hier synonym zu Patient:innenzentrierung verwendet) gezielt zu verbessern, müssen wir ihre Ausprägung reliabel und valide messen können [1]. Nur so kann der Status quo erfasst, Interventionen zur Verbesserung abgeleitet und die Effektivität der Interventionen untersucht werden. Dabei gibt es 3 Perspektiven, aus denen Patient:innenorientierung gemessen werden kann [2]: (1) die Sicht der Behandelnden, (2) die Sicht der Patient:innen und (3) die Sicht von Beobachter:innen. Da das Ziel von Patient:innenorientierung darin besteht, die Patient:innen in den Mittelpunkt der Versorgung zu stellen, ist es sinnvoll, ihre Perspektive einzubeziehen. Bestimmte Aspekte der Patient:innenorientierung – etwa ob Patient:innen sich respektiert fühlten oder ob Informationen verständlich vermittelt wurden – können ausschließlich von den Patient:innen selbst bewertet werden [1, 3]. So argumentieren Tzelepis und ihre Kolleg:innen [4], dass patient:innenberichtete Erhebungsinstrumente am besten geeignet sind, Patient:innenorientierung zu messen, da nur Patient:innen verlässlich einschätzen können, ob ihre Werte, Bedürfnisse und Präferenzen berücksichtigt wurden. Zudem zeigten von diesen 3 Perspektiven patient:innenberichtete Maße die höchste Vorhersagekraft für Behandlungsergebnisse [5].

Zur Erfassung der Erfahrungen von Patient:innen wurden sogenannte patient:innenberichtete Erfahrungsmaße (engl. „patient-reported experience measures“, kurz: PREMs) entwickelt. PREMs konzentrieren sich auf das, was Patient:innen während der Versorgung tatsächlich erlebt haben – also darauf, was passiert ist und wie es passiert ist [6, 7]. Im Fokus stehen konkrete Prozesse der Versorgung: Es wird nicht allgemein nach Zufriedenheit gefragt, sondern nach spezifischen Verhaltensweisen von Behandelnden sowie Prozessen und Abläufen in der erlebten ambulanten oder stationären Versorgung. So wird etwa nicht erhoben, wie zufrieden Patient:innen mit der Kommunikation waren, sondern ob sie die von Behandelnden verwendeten Begriffe verstanden haben oder ob die Behandelnden Blickkontakt aufgebaut haben. Ziel ist es, möglichst objektiv erfassbare Aspekte der Versorgungsqualität aus Patient:innensicht abzubilden. Dieser Fokus erlaubt eine differenzierte Analyse und gezielte Ableitung von Verbesserungsmaßnahmen auf der Verhaltensebene. PREMs sind also Prozessindikatoren interpersoneller Versorgungsqualität, während Zufriedenheitsmaße ein Outcome messen, das das Resultat dieser Erfahrungen widerspiegelt [8].

International finden PREMs in vielfältigen Anwendungsfeldern Verwendung und können auf allen Ebenen des Gesundheitssystems eingesetzt werden: Auf der Mikroebene dienen sie etwa dazu, Rückmeldungen zu individuellen Erfahrungen mit Behandelnden zu geben, auf der Mesoebene ermöglichen sie den Vergleich zwischen Gesundheitseinrichtungen und auf der Makroebene unterstützen sie die Entwicklung systemweiter Strategien zur Verbesserung der Gesundheitsversorgung [3]. Studien zeigen, dass bereits die Rückmeldung und gemeinsame Diskussion der Ergebnisse von PREM-Befragungen positive Veränderungen in der Versorgung bewirken können [9]. Zudem schneiden Behandelnde, die gut über die PREM-Ergebnisse informiert sind, in künftigen Befragungen besser ab [10]. In Ländern wie den USA und dem Vereinigten Königreich werden PREMs systematisch erhoben und die Ergebnisse öffentlich zugänglich gemacht [11]. Dort sind sie ein Bestandteil von Modellen der wertorientierten Gesundheitsversorgung (Value-based Healthcare Models), bei denen nicht nur klinische Ergebnisse mittels Patient-reported Outcome Measures (PROMs), sondern auch Patient:innenerfahrungen mittels PREMs erfasst werden. Positive Erfahrungen können zu Bonuszahlungen an Einrichtungen führen und sollen so die Qualität und Effizienz der Versorgung steigern [11]. So kann die Messung von Patient:innenerfahrungen genutzt werden, um Anreize zu schaffen, die Bedürfnisse von Patient:innen stärker in den Fokus der Versorgung zu rücken.

Dieser Artikel gibt einen Überblick über PREMs in Deutschland. Anhand des Fragebogens zur erlebten Patient:innenorientierung (EPAT; Englisch: Experienced Patient-Centeredness Questionnaire) wird ein Best-Practice-Beispiel für die Entwicklung von reliablen und validen PREMs beschrieben. Schließlich wird der aktuelle Einsatz von PREMs in Deutschland diskutiert.

## Welche PREMs gibt es in Deutschland?

**Abb. 1:**
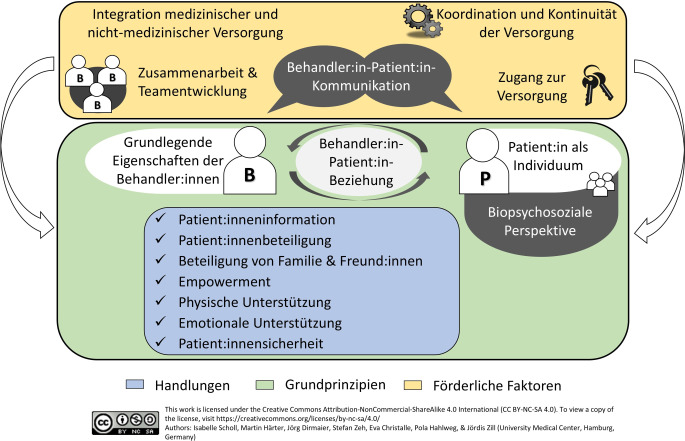
Das integrative Modell für Patient:innenorientierung [14, 24]

In 2022 haben Mihaljevic und seine Kolleg:innen ein umfassendes systematisches Review zu deutschsprachigen PREMs veröffentlicht [12]. Sie fokussierten dabei auf psychometrische Studien zu PREMs, die entweder generisch waren oder sich auf chirurgische Behandlungen oder Krebsbehandlungen bezogen. Sie schlossen PREMs ein, die Patient:innenorientierung multidimensional erfassten und die entweder auf Deutsch entwickelt oder ins Deutsche übersetzt worden waren. Sie bewerteten die Qualität der eingeschlossenen Studien anhand der COSMIN-Qualitätscheckliste (COnsensus-based Standards for the Selection of Health Measurement Instruments; [13]). Außerdem analysierten sie, welche der Dimensionen des integrativen Modells für Patient:innenorientierung [14] von den jeweiligen PREMs erfasst wurde. Dieses Modell basiert auf einem umfassenden systematischen Review, dass 417 Artikel mit Definitionen identifiziert und in einem Modell zusammengefasst hat [14]. Es beschreibt ursprünglich 15 Dimensionen einer patient:innenorientierten Versorgung – eingeteilt in Grundprinzipien (z. B. die Anerkennung von Patient:innen als Individuen), förderliche Faktoren (z. B. durch eine gute Kommunikation zwischen Behandelnden und Patient:innen) sowie Handlungen (z. B. die Beteiligung von Patient:innen in Behandlungsentscheidungen). Einen Überblick über das Modell findet sich in Abb. 1.

Mihaljevic et al. [12] identifizierten 8 generische und 4 krebsspezifische PREMs. Keines der PREMs bezog sich auf chirurgische Behandlungen. Sie stellten fest, dass keines der identifizierten PREMs Patient:innenorientierung vollständig erfasste, und alle zeigten der COSMIN-Checkliste zufolge Mängel. Dennoch gab es dabei einige PREMs, die von den Autor:innen empfohlen wurden. Zwei werden hier genauer vorgestellt.

Im Bereich generischer PREMs tat sich vor allem der Patients’ Experiences Across Health Care Sectors (PEACS; [15]) hervor, der auf Deutsch entwickelt wurde. PEACS ist sowohl für stationäre als auch ambulante Settings einsetzbar, während alle anderen identifizierten generischen PREMs nur für stationäre Settings entwickelt wurden [12]. Es erfasst 13 der 16 Dimensionen des integrativen Modells für Patient:innenorientierung (Abb. 1) mit 59 Items und erhielt gute Ratings für Inhaltsvalidität (außer Vollständigkeit), strukturelle Validität und Reliabilität [12]. Allerdings fehlten Informationen zu interner Konsistenz, Messfehler und Hypothesentestung (Konstruktvalidität; [12]). Im Bereich PREMs spezifisch zur Behandlung von Krebserkrankungen schnitt die deutsche Version des Patient Satisfaction with Cancer-related Care (PSCC‑G; [16]) besonders gut ab. Er wurde vom englischen Original [17] übersetzt und erfasst – trotz seines Namens – Patient:innenerfahrungen zu 10 der 16 Dimensionen von Patient:innenorientierung mit 18 Items [12]. Er erhielt gute Ratings für Inhaltsvalidität, strukturelle Validität, interne Konsistenz, interkulturelle Validität und Hypothesentestung [12]. Es fehlten Informationen zu Reliabilität und Messfehler [12].

Seit der Literatursuche von Mihaljevic et al. wurden weitere PREMs in Deutschland entwickelt und psychometrisch überprüft (z. B. ein Messinstrument zur sektorübergreifenden Evaluation im Bereich psychische Gesundheit [18], ein Instrument zur Messung der Patientensicherheit im Entlassungsprozess [19] und ein Fragebogen zu Erfahrungen in der frühen Geburtsphase [20]). Zudem wurde von uns gemeinsam mit weiteren Kolleg:innen im Rahmen der ASPIRED-Studie (Assessment of Patient Centredness through Patient-reported Experience Measures) der Fragebogen zur erlebten Patient:innenorientierung (EPAT) entwickelt [21]. Er schließt eine wichtige Forschungslücke, da er das erste PREM ist, das alle Dimensionen des integrativen Modells der Patient:innenorientierung (Abb. 1) vollständig erfasst [12] und generisch sowie Setting-übergreifend einsetzbar ist.

## Der Fragebogen zur erlebten Patient:innenorientierung (EPAT)

### Entwicklung und psychometrische Evaluation des EPAT

Der EPAT wurde in einem mehrstufigen Prozess unter Einsatz verschiedener Methoden entwickelt [22]. Der erste Schritt in der Entwicklung eines Fragebogens ist die genaue Definition des Konstrukts – idealerweise unter Einbezug der Zielgruppe, hier also Patient:innen [23]. Dafür wurde das integrative Modell für Patient:innenorientierung als Grundlage genutzt (Abb. 1; [14]). Das Modell wurde in einem Delphi-Verfahren mit Patient:innen vervollständigt sowie die Relevanz der einzelnen Dimensionen aus Sicht der Patient:innen erfasst [24]. Das Ergebnis war, dass eine 16. Dimension (Patient:innensicherheit) ergänzt wurde und alle 16 Dimensionen aus Patient:innensicht als sehr relevant eingestuft wurden [24].

Für jede dieser 16 Dimensionen wurden Items auf Basis von 3 Quellen entwickelt: (1) eine Literaturrecherche, in der PREMs auf Deutsch und Englisch identifiziert wurden, (2) Fokusgruppen mit Patient:innen sowie (3) Expert:innen-Interviews (Key Informant Interviews). Alle Quellen wurden mittels qualitativer Inhaltsanalyse nach Mayring [25] analysiert, daraus Items generiert und schließlich in einem Relevanzrating mit Expert:innen (inklusive Patient:innenvertreter:innen) auf Relevanz und Vollständigkeit überprüft. Schließlich wurde die Verständlichkeit der Items in kognitiven Interviews mit Patient:innen geprüft. Diese 3 Aspekte – Relevanz, Vollständigkeit und Verständlichkeit aus Sicht der Zielgruppe – ergeben gemeinsam Inhaltsvalidität [13]. So wurde sichergestellt, dass die vorläufige Version des EPAT inhaltsvalide ist. Dabei gab es 2 Versionen, eine stationäre mit 121 Items und eine ambulante Version mit 120 Items.

Die psychometrischen Eigenschaften des EPAT wurden in einer Stichprobe von 2024 Patient:innen getestet, die sowohl in ambulanten als auch stationären Settings für eine Erkrankung aus einer der folgenden 4 Erkrankungsgruppen behandelt wurden: (1) Herz-Kreislauf-Erkrankungen, (2) Krebs, (3) muskuloskelettale Erkrankungen und (4) psychische Störungen [26, 27]. Dabei wurden verschiedene Eigenschaften der Items getestet und zur Auswahl der Items für die finale Version des EPAT genutzt (z. B. Akzeptanz der Items [28], Itemschwierigkeit [29] und Item-Total-Korrelationen [30]). Um die Inhaltsvalidität zu gewährleisten – insbesondere die Vollständigkeit der Items –, wurden daten- und theoriegeleitete Ansätze im Auswahlverfahren kombiniert. So wurden für jede der 16 Dimensionen Items anhand der statistischen Kennwerte und Überlegungen hinsichtlich des Inhalts und seiner Relevanz für die Definition der Dimension ausgewählt. Für jede Dimension wurde ein bestes Item ausgewählt, dass sowohl in der ambulanten als auch der stationären Version genutzt wurde. Daraus entstand der EPAT-16, der sich sowohl setting- als auch krankheitsübergreifend einsetzen lässt, um einen Gesamtwert von Patient:innenorientierung mit 16 Items zu bestimmen [26]. Darüber hinaus wurden für jede Dimension 3 weitere Items gewählt. Daraus entstand der EPAT-64, der jede der 16 Dimensionen mit 4 Items erfasst und entsprechend in 2 Versionen vorliegt (ambulant und stationär; [27]). Dabei nutzt er einen modularen Aufbau, das heißt, dass nicht alle 64 Items verwendet werden müssen, sondern stattdessen einzelne Dimensionen zur Messung ausgewählt werden können.

Beide Versionen – EPAT-16 und EPAT-64 – wiesen gute psychometrische Eigenschaften auf. Alle Items zeigten eine hohe Akzeptanz und gute Item-Total-Korrelationen. Die Itemschwierigkeiten ergaben, dass etwa 70 % der Items eine gute Varianz in den Antworten über die gesamte Antwortskala aufwiesen, während bei den anderen Items leichte Deckeneffekte festgestellt wurden. Für beide Versionen konnte sowohl die strukturelle Validität als auch eine hohe Reliabilität bestätigt werden. Alle Hypothesen, die zur Überprüfung der Konstruktvalidität getestet wurden, wurden für beide Versionen bestätigt. Der EPAT-16 ist besonders geeignet, um mit wenigen Items Patient:innenorientierung insgesamt zu messen, während der EPAT-64 es erlaubt, Aussagen über einzelne Dimensionen zu treffen. Eine Übersicht über die Mittelwerte des EPAT-16 in der ambulanten und stationären Evaluationsstichprobe findet sich in Tab. 1. Die Aussagen in den Items wurden auf einer Skala von 1 („trifft überhaupt nicht zu“) bis 6 („trifft völlig zu“) bewertet, wobei ein hoher Wert einer hohen Patient:innenorientierung entspricht. Die Items zu Beteiligung von Familie und Freund:innen sowie Integration medizinischer und nichtmedizinischer Versorgung wurden als am geringsten ausgeprägt bewertet. Besonders gute Bewertungen zeigten sich für die Items zu Behandler:innen-Patient:innen-Beziehung sowie Behandler:innen-Patient:innen-Kommunikation. Detaillierte Ergebnisse finden sich jeweils in den Publikationen zum EPAT-64 [27] und EPAT-16 [26].

**Tab. 1:** Mittelwerte und Standardabweichungen des Fragebogens zur erlebten Patient:innenorientierung EPAT-16 in der Evaluationsstichprobe

Dimension des EPAT-64	Item	Ambulant		Stationär	
		M	SD	M	SD
Grundlegende Eigenschaften der Behandelnden	Meine Behandelnden waren einfühlsam (zum Beispiel sind sie auf meine Gefühle eingegangen, haben Verständnis gezeigt oder haben sich in meine Situation hineinversetzt)	5,1	1,2	5,1	1,2
Behandler:innen-Patient:innen-Beziehung	Ich habe meinen Behandelnden vertraut	5,2	1,1	5,3	1,1
Patient:innen als Individuum	Meine Wünsche, Bedürfnisse und Erwartungen wurden erfragt und in der Behandlung berücksichtigt	4,8	1,4	4,8	1,3
Biopsychosoziale Perspektive	Bei der Behandlung wurde meine gesamte Lebenssituation berücksichtigt (zum Beispiel Beruf, Familie und Freunde, Partnerschaft und Sexualität, Kultur und Religion, Alter oder finanzielle Verhältnisse)	3,9	1,8	3,8	1,8
Behandler:innen-Patient:innen-Kommunikation	Mir wurde genug Zeit gegeben, mein Anliegen und meine Situation zu beschreiben (zum Beispiel bisheriger Verlauf oder aktuelle Symptome)	5,3	1,1	5,2	1,1
Integration medizinischer und nichtmedizinischer Versorgung	Ich wurde gefragt, ob ich ergänzende Angebote nutze oder nutzen möchte (zum Beispiel Selbsthilfegruppen, Beratung, Gesundheitskurse, Alternativmedizin/Komplementärmedizin oder spirituelle Unterstützung/Seelsorge)	3,1	2,0	3,4	2,0
Zusammenarbeit und Teamentwicklung	Die Abläufe innerhalb des Teams waren gut organisiert	5,1	1,0	5,1	1,1
Zugang zur Versorgung	Wenn ich mit einer Ärztin/einem Arzt sprechen wollte, war diese/dieser gut erreichbar	4,7	1,4	4,8	1,2
Koordination und Kontinuität der Versorgung	Mit mir wurde besprochen, ob Folgetermine sinnvoll sind (zum Beispiel zur Nachsorge oder Weiterbehandlung)	5,1	1,4	5,0	1,4
Patient:innensicherheit	Ich wurde ermutigt anzusprechen, wenn mir bei meiner Behandlung Unstimmigkeiten aufgefallen sind	3,9	1,8	4,1	1,7
Patient:inneninformation	Ich habe von den Behandelnden Informationen zu meiner Erkrankung bekommen (zum Beispiel Ursachen, Symptome, Auswirkungen oder Verlauf)	4,6	1,6	4,6	1,5
Patient:innenbeteiligung	Ich war gleichwertige Partnerin oder gleichwertiger Partner auf Augenhöhe mit meinen Behandelnden (zum Beispiel bei Entscheidungen oder Austausch von Informationen)	5,0	1,2	4,8	1,3
Beteiligung von Familie und Freund:innen	Mir wurde erklärt, welche Möglichkeiten es gibt, meine Angehörigen mit in die Behandlung einzubeziehen (zum Beispiel Begleitung zur Behandlung, Teilnahme an Gesprächen oder Unterstützung bei der Einnahme von Medikamenten)	3,1	2,0	3,3	1,9
Empowerment	Ich wurde motiviert, meine Gesundheit zu verbessern, indem ich mein Verhalten ändere (zum Beispiel durch Ernährung, Bewegung, weniger Tabak oder Alkohol)	4,0	1,8	4,0	1,7
Physische Unterstützung	Wenn ich Schmerzen hatte, wurde mir schnell geholfen	4,8	1,4	5,4	1,0
Emotionale Unterstützung	Die Behandelnden sind auf meine Ängste und Sorgen eingegangen (zum Beispiel indem sie Verständnis gezeigt und mir Mut gemacht haben)	4,4	1,7	4,4	1,6

Ergebnisse veröffentlicht in Christalle et al. [26]

*M* Mittelwert, *SD* Standardabweichung

Antwortskala 1 = „trifft überhaupt nicht zu“ bis 6 = „trifft völlig zu“

### Nutzungsmöglichkeiten des EPAT

Alles in allem zeichnet der EPAT sich insbesondere durch folgende Aspekte aus. Alle Schritte von der Item-Entwicklung bis zur Item-Selektion waren theoriegeleitet, indem stets der Inhalt der Items und die Definition des Konstrukts berücksichtigt wurden, was eine hohe Inhaltsvalidität des finalen EPAT sicherstellt. Dabei wurde der Blickwinkel der Zielgruppe – das heißt Patient:innen – konsequent in allen Schritten miteinbezogen, sowohl bei der Definition des Konstrukts als auch der Item-Entwicklung und Item-Selektion.

Weitere Studien zu den psychometrischen Eigenschaften des EPAT sollten die Änderungssensitivität untersuchen, eine wichtige Eigenschaft, um den EPAT zur Evaluation von Interventionen einsetzen zu können. Außerdem sollte die Faktorenstruktur weiter untersucht und die psychometrischen Eigenschaften in anderen Gruppen (z. B. mit anderen Erkrankungen oder in anderen Settings) überprüft werden.

Dank der verschiedenen Versionen und des modularen Aufbaus kann der EPAT flexibel an die Bedürfnisse und Fragestellungen angepasst werden. Diese Flexibilität ermöglicht den Einsatz des EPAT sowohl in der Forschung – etwa zur Erfassung des Status quo der Patient:innenorientierung oder zur Analyse ihrer Zusammenhänge mit anderen Variablen – als auch in der Routineversorgung. In Letzterer kann der EPAT beispielsweise zur kontinuierlichen Rückmeldung an Behandelnde, zur Qualitätssicherung oder für regelmäßiges Monitoring genutzt werden. Gerade für den Einsatz im Versorgungsalltag bietet der EPAT damit ein praktikables Instrument. Ein weiterer Vorteil liegt in der freien Zugänglichkeit des EPAT. Alle zugehörigen Publikationen sind im Open Access verfügbar und die verschiedenen Fragebogenversionen können kostenfrei unter www.uke.de/epat heruntergeladen werden. Außerdem finden Sie die Versionen in den Online-Appendizes 1–4. Die Nutzung erfolgt unter einer Creative-Commons-Lizenz, sodass Einzelpersonen wie auch Einrichtungen – unabhängig von finanziellen Ressourcen oder Trägerschaft – den EPAT einsetzen können. Dies steht im Einklang mit aktuellen Open-Science-Bestrebungen und fördert eine breite Implementierung. Da er ein generischer Fragebogen ist, eignet sich der EPAT auch für bevölkerungsrepräsentative Erhebungen, um ein umfassendes Bild zur Patient:innenorientierung in Deutschland zu gewinnen. Ein Beispiel dafür ist der „TK-Monitor Patientensicherheit 2024“, in dem das Modul „Patientensicherheit“ des EPAT-64 verwendet wurde [31].

## Einsatz von PREMs in Deutschland – Aktueller Stand und Implikationen

**Abb. 2:**
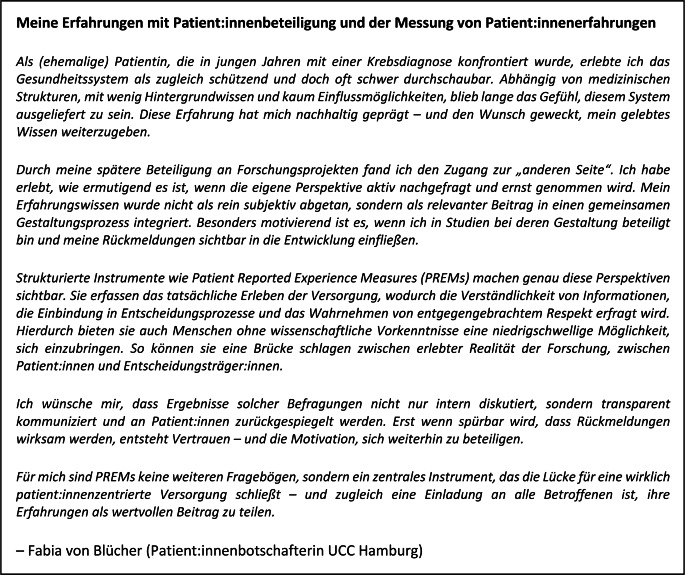
Perspektive einer Patient:innenbotschafterin

Ein zentraler Vorteil generischer PREMs wie des EPAT liegt in ihrer breiten Anwendbarkeit – sowohl für Benchmarking über verschiedene Indikationen und Einrichtungen als auch für öffentliche Bewertungen [6]. Öffentliche Analysen fördern Transparenz und ermöglichen Patient:innen informierte Entscheidungen bei der Wahl von Gesundheitseinrichtungen. Eine Umfrage der Bertelsmann Stiftung zeigte, dass 64 % der Befragten sich bei der Auswahl unzureichend informiert fühlten. 82 % sprachen sich für die öffentliche Bereitstellung von Patient:innenerfahrungen aus [32]. Dieser Wunsch wird auch von der Patient:innenbotschafterin des Universitären Cancer Center (UCC) Hamburg Fabia von Blücher aufgegriffen, die ihre Perspektive zur Messung von Patient:innenerfahrungen in Abb. 2 teilt.

Bei einem solchen systematischen und flächendeckenden Einsatz von PREMs steht Deutschland im internationalen Vergleich – etwa mit den USA, Dänemark oder den Niederlanden – noch am Anfang [33, 34]. Doch es gibt politische und wissenschaftliche Entwicklungen, die den Ausbau dieser Instrumente fördern. Die Gesundheitsministerkonferenz forderte bereits 2018 differenzierte Patient:innenbefragungen als Teil des Qualitätsmanagements in allen Einrichtungen des Gesundheitswesens und die Bereitstellung neutraler und transparenter Ergebnisse als Patient:inneninformation [35]. Seit 2016 legt das Krankenhausstrukturgesetz fest, dass Qualitätsindikatoren bei der Krankenhausplanung miteinbezogen werden müssen [36]. Dafür hat der Gemeinsame Bundesausschuss (G-BA) das Institut für Qualitätssicherung und Transparenz im Gesundheitswesen (IQTIG) beauftragt, Qualitätsindikatoren zu entwickeln [36]. Seit 2022 erfasst das IQTIG dabei auch Patient:innenerfahrungen durch Patient:innenbefragungen im Rahmen der gesetzlichen Qualitätssicherung [37]. Die Entwicklung dieser Patient:innenbefragung wurde vom G‑BA beauftragt [38]. Seitdem gab es mehrere weitere Aufträge vom G‑BA zur Entwicklung von Patient:innenbefragungen. Im Gegensatz zum EPAT verfolgt das IQTIG dabei einen indikationsspezifischen Ansatz. Der mehrstufige Entwicklungsprozess besteht aus Literaturrecherche, Fokusgruppen sowie Interviews mit Patient:innen. Zusätzlich werden sie durch ein Expert:innengremium beraten, zu dem auch Patient:innen gehören [39]. So wird sichergestellt, dass die entwickelten Qualitätsindikatoren für die spezifische Patient:innengruppe relevant sind. Aktuell werden 5 Patient:innenbefragungen zur Qualitätssicherung eingesetzt (zum Beispiel im Bereich ambulante Psychotherapie oder Entlassmanagement). Drei weitere befinden sich in Entwicklung [39]. Die validierten Fragebögen können auf der Webseite des IQTIG beim jeweiligen Qualitätssicherungsverfahren heruntergeladen werden. Darüber hinaus finden auch in anderen Bereichen in Deutschland PREMs zunehmend Einsatz in der Qualitätssicherung. Zum Beispiel hat das Wissenschaftliche Institut der Niedergelassenen Hämatologen und Onkologen (WINHO) seit 2020 seine Datenerhebung explizit auf ein PREM umgestellt [40]. Darüber hinaus förderte der Innovationsfond im Jahr 2021 im Bereich Versorgungsforschung explizit Studien über „Sektorenübergreifende und ambulante PROMs/PREMs“. In diesem Bereich wurden 5 Studien gefördert, wobei aber nur eine – die PROMchronic-Studie – PREMs einsetzt [41]. Ziel dieser Studie ist es, den Nutzen und die Wirksamkeit des bevölkerungsweiten Einsatzes von elektronischen PROMs und PREMs zur Verbesserung der Versorgung chronisch erkrankter Patient:innen in Deutschland zu untersuchen [41]. Doch auch in anderen Themenbereichen fördert der Innovationsfond Forschungsprojekte, die PREMs einsetzen. So verwendet zum Beispiel die NAVIGATION-Studie (Nachhaltig versorgt im gemeindenahen Gesundheitszentrum – Gesundheit im Zentrum) den EPAT, um eine neue Versorgungsform für Menschen in sozial benachteiligten Regionen durch koordinierte Gesundheitsangebote zu evaluieren [42].

Während der EPAT als generisches PREM in Deutschland sehr breit einsetzbar ist [26, 27], stehen mit den PREMs aus den Patient:innenbefragungen des IQTIG indikations- beziehungsweise behandlungsspezifische PREMs zur Verfügung [43]. Allgemein ermöglichen generische PREMs den Vergleich patient:innenbezogener Erfahrungen über verschiedene Krankheitsbilder, Versorgungssettings und Patient:innengruppen hinweg. Sie eignen sich besonders für systemweite Qualitätsvergleiche und gesundheitsökonomische Bewertungen. Ein potenzieller Nachteil liegt jedoch in der möglicherweise begrenzten Sensitivität für krankheitsspezifische Bedürfnisse, Symptome oder Versorgungssituationen [44, 45]. Indikationsspezifische PREMs hingegen erfassen differenzierter Kernerfahrungen innerhalb einer bestimmten Patient:innengruppe und bieten eine höhere inhaltliche Relevanz für klinische Entscheidungsprozesse. Allerdings bedürfen sie entsprechend vieler Ressourcen zur Entwicklung, sind sie in ihrer Anwendbarkeit eingeschränkt und erschweren sektorübergreifende Vergleiche.

Unabhängig von der Wahl des PREMs ist es wichtig, anzumerken, dass die reine Messung von Patient:innenerfahrungen nicht genügt – die Ergebnisse müssen aktiv genutzt werden, um die Versorgungsqualität zu verbessern [46]. Ein möglicher nächster Schritt ist die kontinuierliche Erhebung von PREMs in der Routineversorgung. Hierfür ist es entscheidend, alle Beteiligten – Patient:innen wie Behandelnde – mit in den Umsetzungsprozess einzubeziehen [46]. Sobald PREMs in den Versorgungsalltag integriert sind, sollte der Fokus auf der konkreten Nutzung der Ergebnisse liegen. Beispielsweise können diese in Treffen des Behandlungsteams präsentiert und als Grundlage für gemeinsame Reflexionen über Verbesserungsmöglichkeiten genutzt werden [47]. Zur tieferen Kontextualisierung und besseren Einordnung quantitativer Daten sollte die Erhebung qualitativer Informationen – etwa in Form von Patient:innengeschichten – ergänzt werden [46].

## Fazit

Während PREMs international bereits systematisch zur Qualitätsmessung, zur Patienteninformation und im Rahmen von Value-based Healthcare eingesetzt werden, steht Deutschland noch am Anfang eines flächendeckenden Einsatzes. Doch sowohl auf politischer Ebene als auch in der Wissenschaft und Qualitätssicherung in der Routineversorgung gibt es zunehmende Bemühungen, Patient:innenerfahrungen zu integrieren. Mit dem EPAT liegt nun erstmals ein wissenschaftlich fundiertes, generisches PREM in deutscher Sprache vor, das Patient:innenorientierung umfassend und valide erfasst. Durch seine modulare Struktur und die Eignung für verschiedene Versorgungskontexte – stationär wie ambulant – bietet der EPAT vielfältige Einsatzmöglichkeiten, etwa zur Qualitätssicherung, Forschung oder zur patientenzentrierten Weiterentwicklung der Versorgung. Insgesamt birgt der systematische Einsatz von PREMs in Deutschland noch großes Potenzial, um die Versorgung stärker an den Bedürfnissen der Patient:innen auszurichten und nachhaltig zu verbessern.

## Supplementary Information

Online-Appendix 1: Fragebogen zur erlebten Patientenorientierung (EPAT-16) - ambulante Settings; Online-Appendix 2: Fragebogen zur erlebten Patientenorientierung (EPAT-16) - stationäre Settings; Online-Appendix 3: Fragebogen zur erlebten Patientenorientierung (EPAT-64) - ambulante Settings; Online-Appendix 4: Fragebogen zur erlebten Patientenorientierung (EPAT-64) - stationäre Settings
